# Advanced Imaging Modalities in Gastrointestinal Endoscopy: A Systematic Review of Diagnostic Accuracy and Clinical Impact

**DOI:** 10.7759/cureus.96155

**Published:** 2025-11-05

**Authors:** Anas E Ahmed, Ali M Abudash, Abdul‎rah‎man K Al‎am‎er, Ibrahim N Almuhaihi, Abdulkarim M Alghaithi, Abdullah A Alammar, Maryam K Magfouri, Nasser A Alnasser, Maryam M Abdulaal, Khalid S Altamimi

**Affiliations:** 1 Department of Community Medicine, Jazan University, Jazan, SAU; 2 Department of Emergency Medicine, Sabya General Hospital, Jazan, SAU; 3 College of Medicine, King Faisal University, Al-Ahsa, SAU; 4 College of Medicine, University of Hail, Hail, SAU; 5 College of Medicine, Jazan University, Jazan, SAU; 6 College of Medicine, King Saud University, Riyadh, SAU; 7 College of Medicine, Taibah University, Madinah, SAU

**Keywords:** adenoma detection rate, advanced imaging, artificial intelligence, autofluorescence imaging, blue laser imaging, computer-aided detection, computer-aided diagnosis, gastrointestinal endoscopy, linked color imaging, narrow band imaging

## Abstract

Conventional white light endoscopy (WLE) has limited sensitivity for detecting subtle gastrointestinal lesions, leading to missed diagnoses and interval cancers. Advanced imaging modalities, including narrow band imaging (NBI), linked color imaging (LCI), blue laser imaging (BLI), autofluorescence imaging (AFI), and artificial intelligence (AI)-based systems, have been developed to enhance lesion detection, characterization, and diagnostic confidence. This systematic review, conducted in accordance with the Preferred Reporting Items for Systematic Reviews and Meta-Analyses (PRISMA) guidelines, evaluated the diagnostic accuracy and clinical impact of these technologies compared with WLE and other standard approaches. Comprehensive searches of PubMed, Cochrane Central Register of Controlled Trials (CENTRAL), Scopus, and Web of Science (from inception to September 2025) identified 14,288 records, of which 10,590 unique studies were screened, and 21 met the inclusion criteria. NBI demonstrated moderate-to-high diagnostic accuracy but offered limited improvement over high-definition WLE in adenoma detection. LCI improved sensitivity and lesion visibility across gastric, esophageal, and inflammatory disorders, showing strong performance in early gastric cancer and reflux esophagitis. BLI and BLI-bright enhanced real-time gastric cancer detection and supported colorectal optical diagnosis comparable to NBI. AFI showed inferior performance to dye-based chromoendoscopy in ulcerative colitis surveillance but effectively distinguished reflux phenotypes with high accuracy. AI-based systems consistently increased adenoma detection rate (ADR) and adenomas per colonoscopy (APC) through computer-aided detection (CADe) and achieved clinically actionable accuracy for optical diagnosis in selected settings using computer-aided diagnosis (CADx). Overall, advanced imaging modalities improve lesion detection and diagnostic precision compared with WLE, each offering distinct advantages. LCI and BLI are most effective for early gastric cancer and inflammatory conditions, NBI remains a validated tool for targeted diagnosis, and AI-based systems provide the greatest gains in adenoma yield while showing promise for reliable real-time optical diagnosis. Standardization, multicenter validation, and integration into clinical practice guidelines are essential to optimize their impact on patient outcomes.

## Introduction and background

Gastrointestinal (GI) endoscopy remains the cornerstone of diagnosis and surveillance in digestive diseases, allowing direct mucosal visualization and therapeutic intervention. Conventional white light endoscopy (WLE) is the standard approach; however, its limited ability to detect flat, small, or subtle lesions contributes to missed diagnoses and interval cancers in conditions such as colorectal cancer (CRC), gastric cancer, Barrett’s esophagus, and inflammatory bowel disease [1,2]. These limitations have driven the development of advanced endoscopic imaging modalities aimed at improving lesion detection, characterization, and diagnostic precision.

Advanced imaging technologies such as narrow band imaging (NBI), linked color imaging (LCI), blue laser imaging (BLI), and autofluorescence imaging (AFI) enhance mucosal and vascular contrast to facilitate early recognition of neoplasia and inflammation [1,3,4]. Confocal laser endomicroscopy (CLE) and endocytoscopy further enable in vivo microscopic visualization, providing a “virtual biopsy.” More recently, artificial intelligence (AI)-based systems, including computer-aided detection (CADe) and computer-aided diagnosis (CADx), have shown potential to standardize lesion detection and optical diagnosis across varying levels of operator expertise [5,6]. Collectively, these tools represent a shift from passive visualization toward technology-assisted precision endoscopy [7,8].

Diagnostic performance is commonly assessed using sensitivity, specificity, positive predictive value (PPV), negative predictive value (NPV), and the area under the receiver operating characteristic curve (AUC). Image-enhanced endoscopy has been shown to increase sensitivity for early neoplasia and reduce unnecessary biopsies through improved optical diagnosis. For instance, LCI and BLI enhance the detection of early gastric cancers, while NBI and CADx systems improve the differentiation of diminutive colorectal polyps. However, diagnostic performance varies by lesion characteristics and operator experience, contributing to heterogeneity across studies [5,9-11].

Beyond accuracy, the clinical utility of advanced imaging lies in its impact on quality indicators such as adenoma detection rate (ADR), adenomas per colonoscopy (APC), polyp miss rate, biopsy frequency, and surveillance interval assignment, all of which directly influence patient outcomes. Trials of CADe have shown consistent improvements in ADR and APC without prolonging procedures, while LCI enhances visualization of reflux esophagitis and improves interobserver agreement in eosinophilic esophagitis [1,8,12].

Despite rapid technological progress, uncertainty persists regarding the comparative benefits, limitations, and clinical applicability of these modalities. AFI, for example, performs well in reflux phenotyping but less effectively in ulcerative colitis surveillance, and CADx systems still face challenges with specificity. Heterogeneity across populations, methodologies, and outcome measures complicates interpretation and guideline integration.

## Review

Methodology

Literature Search Strategy

This systematic review followed the Preferred Reporting Items for Systematic Reviews and Meta-Analyses (PRISMA) guidelines [13]. A comprehensive search of PubMed, Cochrane Central Register of Controlled Trials (CENTRAL), Scopus, and Web of Science (WOS) was conducted from inception to September 3, 2025. Search terms combined controlled vocabulary (MeSH/Emtree) and free-text keywords, including “gastrointestinal endoscopy”, “colonoscopy”, “gastroscopy”, “esophagoscopy”, “narrow band imaging (NBI)”, “linked color imaging (LCI)”, “blue laser imaging (BLI)”, “autofluorescence imaging (AFI)”, “confocal laser endomicroscopy (CLE)”, “endocytoscopy”, “artificial intelligence (AI)”, “computer-aided detection (CADe)”, and “computer-aided diagnosis (CADx)”. Boolean operators and database-specific syntax were used to refine results. Filters restricted the search to English-language studies on human subjects. Reference lists of included studies and relevant reviews were manually screened to ensure completeness.

Eligibility Criteria

Eligibility criteria were defined using the PICO (population, intervention, comparison, outcome) framework [14]. Studies were included if they: (1) involved patients undergoing GI endoscopy for screening, surveillance, or diagnosis; (2) evaluated advanced imaging modalities (NBI, LCI, BLI/BLI-bright, AFI, CLE, endocytoscopy, or AI-based systems such as CADe/CADx); (3) compared these modalities with standard WLE, expert assessment, or alternative imaging methods; and (4) reported diagnostic accuracy outcomes (sensitivity, specificity, accuracy, PPV, NPV, AUC) and/or clinical impact outcomes (ADR, APC, biopsy rate, or surveillance interval agreement). Excluded were reviews, meta-analyses, case reports, editorials, conference abstracts, non-English papers, and studies lacking extractable diagnostic or clinical data.

Study Selection

Two reviewers independently screened all titles and abstracts, followed by full-text assessment of eligible studies. Discrepancies were resolved through discussion or, if needed, adjudication by a third reviewer. Exclusion at the full-text stage was typically due to inappropriate study design, absence of advanced imaging intervention, or incomplete reporting of diagnostic outcomes.

Data Extraction

Two reviewers independently extracted data using a standardized form. Variables included study ID (author, year), country, design, participant characteristics, imaging modality, comparator, lesion or disease type, outcomes related to diagnostic accuracy (sensitivity, specificity, accuracy, AUC, PPV, NPV), and clinical impact (ADR, APC, miss rate, biopsy reduction, surveillance interval assignment). Disagreements were resolved by consensus with a third reviewer.

Quality Appraisal

Methodological quality was assessed independently by two reviewers using the QUADAS-2 (Quality Assessment of Diagnostic Accuracy Studies 2) tool [15]. Risk of bias and applicability were evaluated across four domains: patient selection, index test, reference standard, and flow/timing. Each domain was rated as low, high, or unclear risk, and disagreements were resolved by discussion and consensus.

Results

Study Selection

**Figure 1:**
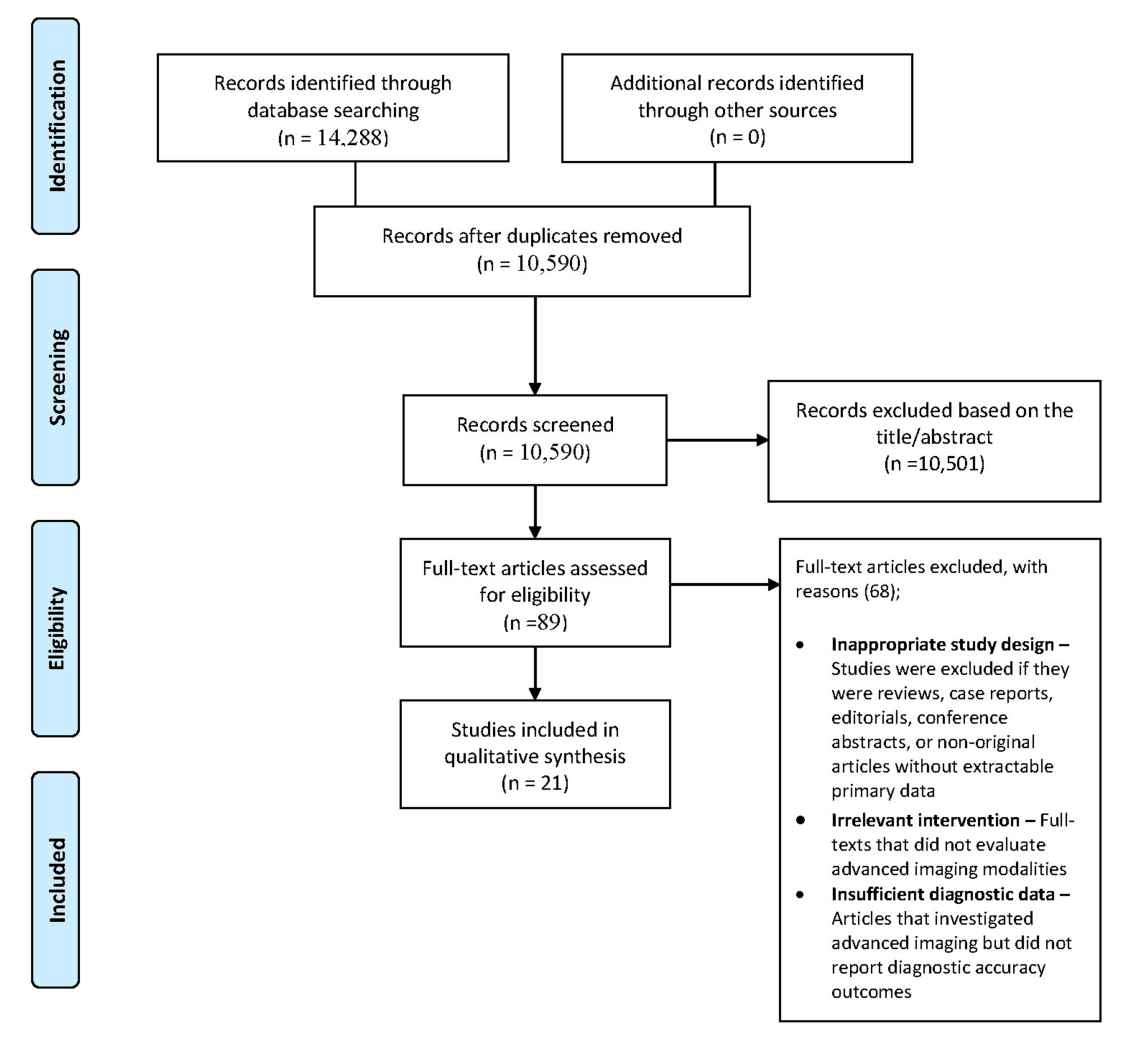
Flow diagram of the study selection process.

A total of 14,288 records were identified across PubMed (6,951), Cochrane Library (1,195), Scopus (3,758), and Web of Science (2,384). After removing duplicates, 10,590 unique studies were screened. Following title and abstract screening, 10,501 records were excluded, leaving 89 full-text articles for eligibility assessment. Of these, 68 were excluded for inappropriate design, irrelevant intervention, or insufficient diagnostic data. Ultimately, 21 studies [1-12,16-24] met the inclusion criteria and were analyzed for diagnostic accuracy and clinical impact of advanced imaging modalities in GI endoscopy (Figure 1).

Study Characteristics

The 21 included studies encompassed diverse designs, populations, and GI conditions (Table 1). Several large multicenter trials evaluated AI systems and optical imaging technologies. Nemoto et al. [8] showed that a ResNet-50-based CADx model achieved accuracy comparable to expert endoscopists for early CRC, while Haruma et al. [9] and Khurelbaatar et al. [5] demonstrated that LCI significantly improved the detection of upper GI neoplasia and early gastric cancer compared with WLE. Houwen et al. [19] validated the POLAR CADx system in fecal immunochemical test (FIT)-positive patients, reporting near-expert diagnostic performance.

**Table 1:** Summary of key studies evaluating advanced endoscopic imaging modalities and artificial intelligence systems. AFI = autofluorescence imaging; AI = artificial intelligence; AI-H = AI-assisted hybrid mode; AMR = adenoma miss rate; APC = adenomas per colonoscopy; ASGE = American Society for Gastrointestinal Endoscopy; AUC = area under the receiver operating characteristic curve; BLI = blue laser imaging; BLI-bright = high-brightness BLI mode; CADe = computer-aided detection; CADx = computer-aided diagnosis system; CE = chromoendoscopy; CI = confidence interval; CRC = colorectal cancer; ΔE* = color difference value in CIE Lab* space; EGC = early gastric cancer; EPMR = endoscopic piecemeal mucosal resection; ERE = erosive reflux esophagitis; FH = functional heartburn; GI = gastrointestinal; HDWL = high-definition white light; HD-WLE = high-definition white-light endoscopy; HGD = high-grade dysplasia; HGIN = high-grade intraepithelial neoplasia; HP = hyperplastic; HRA-F = high-risk assessment form; ICC = intraclass correlation coefficient; IM = intestinal metaplasia; IN = intraepithelial neoplasia; IVH = intraventricular hemorrhage; LA = Los Angeles classification system; LCI = linked color imaging; LGD = low-grade dysplasia; MCE = minimal change esophagitis; NBI = narrow band imaging; NERD = non-erosive reflux disease; NPV = negative predictive value; OR = odds ratio; PIVI = Preservation and Incorporation of Valuable Endoscopic Innovations (ASGE benchmark); PMR = polyp miss rate; PPI = proton pump inhibitor; PPV = positive predictive value; PSC = primary sclerosing cholangitis; RCT = randomized controlled trial; RE = reflux esophagitis; RR = relative risk; SCC = squamous cell carcinoma; SGLT2 = sodium-glucose cotransporter 2; SM = submucosal invasion; SPS = serrated polyposis syndrome; SSL = sessile serrated lesion; Tis = carcinoma in situ; T1a/T1b = early invasive carcinoma (submucosal depth classification); UC = ulcerative colitis; WLE = white light endoscopy; WLI = white light imaging.

Study ID	Country	Study design	Population characteristics	Intervention/imaging modality details	Lesion/disease characteristics	Diagnostic accuracy outcomes	Clinical impact outcomes
Glissen Brown et al. (2022) [1]	United States (4 centers)	Prospective, multicenter, single-blind tandem colonoscopy RCT	223 patients (mean age = 61; 45.3% female); indications: CRC screening = 59.6%, surveillance = 40.4%	Deep-learning CADe (EndoScreener, Wision AI, SegNet); Olympus CLV-190; CADe-first vs. HDWL-first randomization	285 polyps CADe-first; 264 HDWL-first; 169 adenomas, 55 HP, 14 SSLs; mostly <10 mm	AMR: 20.1% CADe vs. 31.3% HDWL (p = 0.025); PMR: 20.7% vs. 33.7% (p = 0.0007); SSL miss: 7.1% vs. 42.1% (p = 0.048)	CADe reduced adenoma/SSL miss rates and improved APC without increasing non-neoplastic resections
Djinbachian et al. (2024) [2]	Canada (Montreal Univ. Hosp. Center)	Single-center RCT (non-inferiority)	467 patients randomized (238 autonomous AI, 229 AI-H); mean age = 64; 49% female	Autonomous AI (Fujifilm CADeye, Eluxeo 7000) vs. AI-assisted optical diagnosis	318 polyps AI, 404 AI-H; histology: adenomas 62%, HP = ~20%, SSL = 3–4%	Accuracy: 77.2% (AI) vs. 72.1% (AI-H) (p = 0.86); Sens: 84.8% vs. 83.6%; Spec: 64.4% vs. 63.8%; NPV: 63.0% vs. 71.0%	Surveillance interval agreement: 91.5% AI vs. 82.1% AI-H (p = 0.016); both met PIVI 2, only AI met PIVI 1 benchmarks
Dohi et al. (2019) [3]	Japan (Kyoto Prefectural Univ. of Medicine & Asahi Univ. Hospital)	Prospective RCT	629 high-risk patients (atrophic gastritis/IM or post-EGC resection); median age = 73; 65% male	Blue laser imaging-bright (BLI-bright) vs. WLI; Fujifilm LASEREO EG-L590ZW/EG-L600ZW	53 new gastric cancers (mostly depressed-type ≤20 mm T1a adenocarcinomas)	Real-time EGC detection: BLI-bright 93.1% vs. WLI 50.0% (p = 0.001); higher for H. pylori-eradicated stomachs	BLI-bright improved EGC detection, rescuing missed lesions; all detected cancers were treated curatively
Riu Pons et al. (2020) [4]	Spain (Hospital del Mar, Barcelona)	Randomized cross-over tandem colonoscopy trial	41 patients with prior serrated lesions (≥1 ≥10 mm or ≥3 >5 mm, proximal to sigmoid); mean age = 59.6; 53.7% male; smokers = 34.2%	Narrow-band imaging (NBI) vs. High-definition WLE (HD-WLE); Olympus EVIS EXERA III CV-190 scopes; five experienced endoscopists (>4000 colonoscopies each)	246 polyps detected, 228 analyzed: 113 hyperplastic (49.6%), 28 SSLs (12.3%), 57 adenomas (25%), 29 normal (12.7%), 1 lipoma; 34 relevant serrated lesions proximal to sigmoid	Detection rates: NBI 47.4% vs. HD-WLE 51.9% (OR: 0.84, 95% CI: 0.37–1.91); polyp miss rate: NBI 21.3% vs. HD-WLE 26.1%	Nine patients (22%) reclassified as SPS after reassessment; suggested shorter surveillance intervals may improve SPS diagnosis
Khurelbaatar et al. (2022) [5]	Japan (Jichi Medical University)	Retrospective analysis of prospectively collected video data	81 patients; median age = 70 (range = 44–89); 64 M/17 F; 52 early gastric cancer (EGC), 29 atrophic gastritis/non-malignant lesions	LCI vs. WLI using ultrathin (EG-L580NW, 5.9 mm) and standard (EG-L590WR, 9.6 mm) endoscopes; videos reviewed by 3 expert endoscopists	52 EGC lesions (proximal 5, middle 14, distal 33); morphology: elevated 12, flat 4, depressed 36; histology: 48 differentiated, 4 undifferentiated; depth: M 41, SM1 4, SM2 7	Sensitivity: ultrathin WLI 66.0%, ultrathin LCI 80.3%, standard WLI 69.9%, standard LCI 84.0%. Specificity: ultrathin WLI 67.8%, ultrathin LCI 59.3%, standard WLI 59.8%, standard LCI 50.6%; κ 0.28–0.51	Ultrathin LCI significantly improved visibility and color contrast; higher diagnostic yield regardless of H. pylori status, lesion size, or location
Vleugels et al. (2018) [6]	Netherlands (5 centers) & UK	International multicenter RCT	210 UC patients (extensive ≥8 y or left-sided ≥15 y); mean age 56.1; 41.9% female; median duration 21 y; 16% prior dysplasia; 18% PSC	Autofluorescence imaging (AFI) vs. chromoendoscopy (CE); Olympus CFH240AZL/I; Lucera Elite processors; preceded by HD-WLE	52 dysplastic lesions in 34 patients; flat/elevated (Paris IIa/IIb); most in inflamed segments	Dysplasia detection: AFI 12.4% vs. CE 19.1% (RR: 0.65, 80% CI: 0.43–0.99); mean lesions/patient: 0.13 vs. 0.37 (p = 0.01)	CE detected more dysplastic/neoplastic lesions but required longer withdrawal times; AFI was faster but not non-inferior
Luo et al. (2016) [7]	China (PLA General Hospital, Beijing)	Prospective observational study	84 patients (mean age = 49 ± 12.6; 39 M/45 F) with reflux >6 months; normal WLI findings	Autofluorescence imaging (AFI) vs. pH-impedance + PPI test	NERD vs. FH; defined by pH-impedance and PPI response	AFI: Sensitivity: 90.5%, Specificity: 90.0%, Accuracy: 90.5%, PPV: 98.5%, NPV: 56.3%	AFI may help distinguish NERD from FH without invasive testing
Nemoto et al. (2023) [8]	Japan (10 academic hospitals)	Multicenter retrospective diagnostic study	1470 patients; mean age = 71 (range = 35–92); 62.2% male; 1513 early-stage CRC lesions; training (n=1110), testing (n=403)	Computer-aided diagnosis (CADx) system using non-magnified white light endoscopy (WLE) images; ResNet-50 deep learning backbone; compared with expert and trainee endoscopists	Lesions: Tis (71%), T1a (9.6%), T1b (19.4%); excluded poor-quality images, lesions ≥5 cm, pedunculated lesions; colon and rectum	CADx (per-lesion, at 90% cutoff): Sensitivity: 59.8%, Specificity: 94.4%, Accuracy: 87.3%, PPV: 73.1%, NPV: 90.2%, AUC: 85.1%. Equivalent to experts, superior to trainees	CADx avoided unnecessary surgery in Tis/T1a cases by high specificity, a potential supportive tool for trainees and centers lacking advanced imaging modalities
Haruma et al. (2022) [9]	Japan (19 hospitals)	Exploratory post-hoc sub-analysis of the multicenter RCT “LCI-FIND”	Total randomized ≈1504; analyzed: WLI (n=751), LCI (n=750); ultraslim (n=223), standard (n=1279); median age = 71–73 y; 76–81% male; sedation: 12.6% (ultraslim) vs. 39.2% (standard)	Linked color imaging (LCI) vs. white light imaging (WLI); Fujifilm LASEREO systems; ultraslim scopes EG-L580NW/NW7; standard EG-L590WR/EG-L600WR7	Upper GI (pharynx, esophagus, stomach); neoplasia defined (e.g., esophageal IN/carcinoma, gastric adenoma/carcinoma); lesion-level breakdown by site/size/morphology reported	Ultraslim, primary mode (patient-level): LCI 17.0% vs. WLI 7.7% (RR: 2.21; adj OR: 2.46). Standard: LCI: 6.5% vs. WLI 4.3% (RR: 1.53; adj OR: 1.57)	Higher tumor yield with LCI (ultraslim 42.6% vs. 32.3% WLI); advantage for esophageal/depressed lesions; lower sedation need with ultraslim scopes
Riu Pons et al. (2018) [10]	Spain (Hospital del Mar, Barcelona)	Prospective single-blind observational study	111 patients (112 post-EPMR scars); mean age = 67.7; 57% male	NBI vs. WLE; Olympus EVIS EXERA III CV-190	Polypectomy scars after EPMR (median baseline polyp size = 20 mm; mostly adenomas with LGD/HGD)	WLE - Sensitivity: 78.9%, Specificity: 84.2%, Accuracy: 81.6%; NBI - Sensitivity: 85.0%, Specificity: 77.1%, Accuracy: 81.1%	NBI may slightly improve residual neoplasia detection, but biopsies are still required
Takeda et al. (2020) [11]	Japan (Juntendo Tokyo Koto Geriatric Medical Center)	Retrospective, single-center clinical study	142 patients with reflux esophagitis; mean age = 67.1 (27–89); 63 M/79 F; LA grades: M (52), A (52), B (24), C (11), D (3); hiatus hernia: 76.1%; H. pylori: 10 +, 91 –, 41 post-eradication	LCI vs. WLI vs. BLI; Fujifilm LASEREO systems (EG-L590WR, EG-L600WR7, EG-L600ZW); 10 endoscopists (5 experts, 5 trainees)	RE classified by modified LA system; MCE and ERE (grades A–D)	Visibility improved with LCI in 28.2% of RE cases (MCE: 19.2%, ERE: 33.3%); no improvement with BLI (0%); ICC LCI 0.57 (MCE), 0.73 (ERE); ΔE*: WLI 12.3 vs. LCI 22.7 (MCE)	LCI improved subjective/objective visibility of RE, especially ERE; may enhance diagnostic confidence and reduce variability
Chang et al. (2022) [12]	Thailand (Siriraj & Hatyai Hospitals)	Prospective RCT (non-inferiority)	362 enrolled, 164 analyzed (91 NBI, 73 BLI); mean age = 61.7; 50.6% male	BLI-bright vs. NBI without magnification; Olympus PCF-H190DL (NBI) vs. Fujifilm EC-L590ZW (BLI)	324 diminutive colorectal polyps ≤5 mm; mean size = 3.5 mm; ~60% proximal, ~40% rectosigmoid	Accuracy: BLI 86.4%, NBI 90.1%; Sensitivity: BLI 98.3%, NBI 99.1%; Specificity: BLI 55.6%, NBI 67.4%; κ 0.80–0.86	Both achieved ≥90% NPV in rectosigmoid; support ASGE “diagnose-and-leave” strategy; high interobserver agreement
Yamasaki et al. (2015) [24]	Japan (Tsuyama Chuo Hospital)	Prospective RCT	51 high-risk patients for esophageal cancer (HRA-F ≥6 or past head/neck cancer); mean age = ~60; 50 M/1 F	Magnifying NBI vs. Lugol Chromoendoscopy; Olympus GIF-H260Z; EVIS LUCERA	Target: superficial esophageal SCC; biopsies for abnormal NBI areas or Lugol-voiding lesions >5 mm	Both identified SCC/HGIN; Lugol had more biopsy targets (6 vs. 1); 1 SCC confirmed (Lugol)	NBI reduced discomfort and procedure time; recommended for follow-up screening due to better tolerability

Among optical modalities, NBI provided similar detection to high-definition white-light endoscopy (HD-WLE) but slightly lower miss rates for serrated lesions [4]. LCI improved visibility and interobserver agreement in reflux esophagitis [11] and eosinophilic esophagitis [13]. AFI underperformed compared to chromoendoscopy in ulcerative colitis surveillance [17], while BLI and BLI-bright enhanced early gastric cancer detection and matched NBI for colorectal optical diagnosis [12,17]. Additional studies confirmed LCI’s superiority in visualizing *Helicobacter pylori*-related gastritis [18] and highlighted modality-specific advantages across disease contexts [3,9,11,12].

Several randomized controlled trials evaluated AI systems. Repici et al. [20] and Shaukat et al. [21] showed that CADe significantly increased ADR and APC without extending procedure time. Djinbachian et al. [15] found that autonomous CADx improved surveillance interval alignment versus AI-assisted diagnosis, while Glissen Brown et al. [1] reported reduced adenoma and sessile serrated lesion miss rates with CADe.

Quality Assessment

Overall methodological quality was high, with most studies rated as low risk of bias using QUADAS-2 (Table 2). Representative patient cohorts, blinded assessments, and standardized protocols were common [4,8,9,12,19]. Only one study (Khurelbaatar et al. [5]) showed unclear bias due to retrospective video review. Applicability concerns were minimal, with populations and technologies closely aligned to clinical practice, particularly in colorectal screening [1,8,20], gastric cancer surveillance [3,17], and upper GI inflammatory conditions [11].

**Table 2:** Quality assessment of included studies using the QUADAS-2 tool. Risk of bias and applicability concerns were evaluated across four domains: patient selection, index test, reference standard, and flow & timing. Each domain was assessed for both risk of bias (low, unclear, high) and applicability (low, unclear, high). QUADAS-2 = Quality Assessment of Diagnostic Accuracy Studies 2; AFI = autofluorescence imaging; RCT = randomized controlled trial; WLE = white light endoscopy; LCI = linked color imaging; BLI = blue laser imaging; NBI = narrow band imaging; HDWL = high-definition white light; CADe = computer-aided detection; CADx = computer-aided diagnosis; AI = artificial intelligence; EMR = endoscopic mucosal resection; EGC = early gastric cancer; UC = ulcerative colitis; GERD = gastroesophageal reflux disease; NERD = non-erosive reflux disease; PPI = proton pump inhibitor; NICE: National Institute for Health and Care Excellence; CRC = colorectal cancer; ESD = endoscopic submucosal dissection; IEE = image enhanced endoscopy. Bias = potential for systematic error affecting study validity. Applicability = relevance of study population, index test, or reference standard to the review question.

Study ID	Patient selection (bias)	Index test (bias)	Reference standard (bias)	Flow & timing (bias)	Patient selection (applicability)	Index test (applicability)	Reference standard (applicability)
Glissen Brown et al. [1]	Low – Multicenter RCT, diverse US population, randomized	Low – CADe vs. HDWL, standardized tandem protocol	Low – Pathologists blinded, histology reference	Low – Same-session tandem exams, minimal exclusions	Low – US CRC screening/surveillance population	Low – Deep-learning CADe index test is highly relevant	Low – Pathology appropriate gold standard
Djinbachian et al. [2]	Low – Randomized trial, clear inclusion/exclusion	Low – Autonomous AI vs. AI-assisted human optical diagnosis applied under blinding	Low – Histopathology reference	Low – All patients analyzed, same session	Low – Screening population representative	Low – AI-assisted index test relevant	Low – Pathology robust
Dohi et al. [3]	Low – Randomized multicenter trial, clear eligibility	Low – Randomization between WLI and BLI-bright, standardized criteria	Low – Biopsy/ESD pathology, blinded central review	Low – Both modalities applied in the same session	Low – High-risk gastric cancer surveillance population	Low – BLI-bright index test fully relevant	Low – Pathology gold standard robust
Riu Pons et al. [4]	Low – Consecutive high-risk patients, clear inclusion/exclusion	Low – Randomized order, same endoscopist, blinded	Low – Histopathology by blinded pathologists	Low – Same-day tandem colonoscopy	Low – Representative serrated lesion population	Low – Relevant IEE modalities	Low – Pathology robust
Khurelbaatar et al. [5]	Low – Consecutive EGC/atrophy cases, exclusions justified	Unclear – Video-based retrospective analysis, possible observer bias	Low – Histopathology confirmation	Low – All lesions analyzed; 90s video clips may limit real-time flow	Low – Population with EGC/atrophy representative	Low – Ultrathin LCI applicable	Low – Histopathology gold standard
Vleugels et al. [6]	Low – Multicenter RCT, inclusion/exclusion clearly described	Low – Chromoendoscopy vs. autofluorescence imaging applied consistently	Low – Histopathology as reference standard	Low – Same-session exams, minimal exclusions	Low – Long-standing UC population representative	Low – IEE modalities relevant	Low – Pathology appropriate
Luo et al. [7]	Low – Prospective, well-defined reflux cohort, exclusions reported	Low – AFI performed systematically, with simple visual criteria	Low – Dual reference: pH/impedance monitoring + PPI response	Low – All patients completed the index and reference tests	Low – GERD/NERD patient population relevant	Low – AFI index modality applicable	Low – Combined physiologic + clinical standard robust
Nemoto et al. [8]	Low – Consecutive multicenter dataset, exclusions justified	Low – CADx tested prospectively on test set, endoscopists blinded	Low – Histopathology gold standard	Low – All patients analyzed, clear timing	Low – Representative early CRC cohort	Low – CADx WLE relevant	Low – Histopathology appropriate
Haruma et al. [9]	Low – Randomized, broad inclusion	Low – LCI vs. WLE applied consistently	Low – Histopathology used	Low – Same-day procedures, no dropouts	Low – Screening population applicable	Low – LCI index test relevant	Low – Pathology valid
Riu Pons et al. [10]	Low – Consecutive post-EMR patients, randomized order	Low – WLE and NBI applied under standardized protocol	Low – Biopsy/histopathology for all scars	Low – Same session, no attrition	Low – Post-EMR surveillance cohort relevant	Low – NBI/WLE directly relevant	Low – Pathology robust
Takeda et al. [11]	Low – Prospective cohort, clear inclusion/exclusion	Low – LCI applied systematically	Low – Histopathology reference standard	Low – Same-session exams	Low – Reflux esophagitis population representative	Low – LCI relevant	Low – Histopathology robust
Chang et al. [12]	Low – RCT, randomization described	Low – BLI vs. NBI compared under blinding, standardized NICE classification	Low – Histology by blinded pathologists	Low – Immediate resection, no flow issues	Low – Applicable colorectal screening/surveillance population	Low – IEE techniques relevant	Low – Histopathology robust
Yamasaki et al. [24]	Low – Prospective, consecutive patients, tolerability study	Low – Magnifying NBI applied consistently	Low – Histopathology used	Low – All patients included, same session	Low – Esophageal cancer screening population	Low – Index test relevant	Low – Pathology appropriate

Narrow Band Imaging (NBI)

NBI consistently demonstrated moderate-to-high diagnostic accuracy across upper and lower GI applications but offered limited improvement over HD-WLE in detection metrics. Riu Pons et al. [4] reported similar serrated lesion detection between NBI and HD-WLE but slightly lower miss rates with NBI (25.0% vs. 28.6%). Its main value lies in enhanced microvascular visualization, enabling targeted biopsies and improved lesion characterization in Barrett’s esophagus and post-resection scars [4,10]. While its impact on ADR is inconsistent, NBI remains a validated benchmark for optical diagnosis.

Linked Color Imaging (LCI)

LCI consistently enhanced lesion visibility and diagnostic performance, particularly in the upper GI tract. In a multicenter RCT, Haruma et al. [9] found LCI doubled neoplasia detection in ultraslim scopes (17.0% vs. 7.7% with WLE). Khurelbaatar et al. [5] confirmed superior sensitivity for early gastric cancer with both ultrathin and standard scopes (80-84% vs. 66-70% for WLE). Takeda et al. [11] showed improved visualization in 28% of reflux esophagitis cases, while Abe et al. [16] reported better interobserver agreement for eosinophilic esophagitis. Collectively, LCI improves sensitivity, color contrast, and diagnostic consistency across upper GI disorders.

Blue Laser Imaging (BLI and BLI-Bright)

BLI enhances mucosal contrast and supports accurate real-time diagnosis. Dohi et al. [17] found that magnifying BLI achieved higher accuracy than WLE in early gastric cancer (92% vs. 72%), and Dohi et al. [3] showed that BLI-bright nearly doubled first-pass detection rates (93% vs. 50%) in high-risk gastric surveillance. For colorectal polyps, BLI performed comparably to NBI (accuracy 86-90%) and maintained high interobserver agreement [12]. BLI excels in microvascular assessment for neoplasia, while LCI remains superior for inflammatory lesions [13].

Autofluorescence Imaging (AFI)

AFI differentiates neoplastic from normal tissue using fluorescence contrast. In ulcerative colitis surveillance, AFI detected fewer dysplastic lesions than chromoendoscopy, confirming the latter’s continued superiority. However, AFI successfully distinguished non-erosive reflux disease from functional heartburn with ~90% sensitivity and specificity [2], supporting its potential role in functional esophageal disorders.

Artificial Intelligence-Based Systems (CADe/CADx)

CADe consistently improved adenoma yield and reduced miss rates across RCTs. ADR increased from 40% to 55% in the study by Repici et al. [20], with a 46% rise in APC. Similar results were reported by Shaukat et al. [21] and Glissen Brown et al. [1]. CADx systems achieved accuracy approaching expert levels: POLAR (Houwen et al. [19]) reached 80% accuracy; Nemoto et al. [8] reported 87% accuracy and 94% specificity; and Djinbachian et al. [2] demonstrated improved adherence to surveillance guidelines using autonomous CADx. AI thus provides consistent gains in lesion detection and near-clinical-grade performance for optical diagnosis.

Cross-Modality Synthesis

Color-enhancement modalities (LCI, BLI, and NBI) consistently outperformed HD-WLE in lesion visibility and diagnostic accuracy, with LCI excelling in gastric and esophageal neoplasia and BLI in early gastric cancer and colorectal optical diagnosis [3,9,11,12]. AFI offered niche utility for reflux phenotyping but lagged behind chromoendoscopy in ulcerative colitis [7]. AI integration yielded the largest diagnostic improvements, with CADe enhancing detection and CADx approaching reliable real-time histologic classification [8,20]. Together, these advances illustrate a progressive shift toward technology-assisted precision endoscopy.

## Conclusions

This systematic review underscores the substantial advances achieved with advanced imaging modalities in gastrointestinal endoscopy. NBI, LCI, and BLI/BLI-bright consistently enhance mucosal visualization and diagnostic accuracy compared with conventional WLE, though their benefits vary by lesion type and clinical context. AFI offers limited value in ulcerative colitis surveillance but shows potential in functional esophageal disorders. AI-based systems, particularly CADe, reliably increase adenoma yield and reduce miss rates, while CADx is approaching expert-level accuracy in real-time optical diagnosis. Collectively, these technologies advance diagnostic precision, reduce inter-operator variability, and support the evolution toward precision endoscopy. However, heterogeneity in study design, modality application, and operator expertise still limits broad clinical standardization. Future research should prioritize large multicenter validation, cross-platform AI integration, and long-term outcome analyses to define cost-effectiveness and establish standardized implementation pathways.

## References

[1] Glissen Brown JR, Mansour NM, Wang P (2022). Deep learning computer-aided polyp detection reduces adenoma miss rate: a United States multi-center randomized tandem colonoscopy study (CADeT-CS Trial). Clin Gastroenterol Hepatol.

[2] Djinbachian R, Haumesser C, Taghiakbari M (2024). Autonomous artificial intelligence vs artificial intelligence-assisted human optical diagnosis of colorectal polyps: a randomized controlled trial. Gastroenterology.

[3] Dohi O, Yagi N, Naito Y (2019). Blue laser imaging-bright improves the real-time detection rate of early gastric cancer: a randomized controlled study. Gastrointest Endosc.

[4] Riu Pons F, Andreu M, Naranjo D (2020). Narrow-band imaging and high-definition white-light endoscopy in patients with serrated lesions not fulfilling criteria for serrated polyposis syndrome: a randomized controlled trial with tandem colonoscopy. BMC Gastroenterol.

[5] Khurelbaatar T, Miura Y, Osawa H (2022). Improved detection of early gastric cancer with linked color imaging using an ultrathin endoscope: a video-based analysis. Endosc Int Open.

[6] Vleugels JLA, Rutter MD, Ragunath K (2018). Chromoendoscopy versus autofluorescence imaging for neoplasia detection in patients with longstanding ulcerative colitis (FIND-UC): an international, multicentre, randomised controlled trial. Lancet Gastroenterol Hepatol.

[7] Luo X, Guo XX, Wang WF, Peng LH, Yang YS, Uedo N (2016). Autofluorescence imaging endoscopy can distinguish non-erosive reflux disease from functional heartburn: a pilot study. World J Gastroenterol.

[8] Nemoto D, Guo Z, Katsuki S (2023). Computer-aided diagnosis of early-stage colorectal cancer using nonmagnified endoscopic white-light images (with videos). Gastrointest Endosc.

[9] Haruma K, Kato M, Kawada K (2022). Diagnostic ability of linked color imaging in ultraslim endoscopy to identify neoplastic lesions in the upper gastrointestinal tract. Endosc Int Open.

[10] Riu Pons F, Andreu M, Gimeno Beltran J (2018). Narrow band imaging and white light endoscopy in the characterization of a polypectomy scar: a single-blind observational study. World J Gastroenterol.

[11] Takeda T, Asaoka D, Abe D (2020). Linked color imaging improves visibility of reflux esophagitis. BMC Gastroenterol.

[12] Chang A, Munjit P, Sriprayoon T, Pongpaibul A, Prachayakul V (2022). Comparison of blue laser imaging and narrow band imaging for the differentiation of diminutive colorectal polyps: a randomized controlled trial. Surg Endosc.

[13] Page MJ, McKenzie JE, Bossuyt PM (2021). The PRISMA 2020 statement: an updated guideline for reporting systematic reviews. BMJ.

[14] Schardt C, Adams MB, Owens T, Keitz S, Fontelo P (2007). Utilization of the PICO framework to improve searching PubMed for clinical questions. BMC Med Inform Decis Mak.

[15] Whiting PF, Rutjes AW, Westwood ME (2011). QUADAS-2: a revised tool for the quality assessment of diagnostic accuracy studies. Ann Intern Med.

[16] Abe Y, Sasaki Y, Yagi M (2023). Linked color imaging improves the diagnostic accuracy of eosinophilic esophagitis. DEN Open.

[17] Dohi O, Yagi N, Majima A (2017). Diagnostic ability of magnifying endoscopy with blue laser imaging for early gastric cancer: a prospective study. Gastric Cancer.

[18] Dos Santos CE, Moreira H, Pereira-Lima JC (2017). Hyoscine butylbromide for colorectal polyp detection: prospective, randomized, placebo-controlled trial. Clinics (Sao Paulo).

[19] Houwen BB, Hazewinkel Y, Giotis I (2023). Computer-aided diagnosis for optical diagnosis of diminutive colorectal polyps including sessile serrated lesions: a real-time comparison with screening endoscopists. Endoscopy.

[20] Repici A, Badalamenti M, Maselli R (2020). Efficacy of real-time computer-aided detection of colorectal neoplasia in a randomized trial. Gastroenterology.

[21] Shaukat A, Lichtenstein DR, Somers SC (2022). Computer-aided detection improves adenomas per colonoscopy for screening and surveillance colonoscopy: a randomized trial. Gastroenterology.

[22] Suzuki H, Yamamura T, Nakamura M (2020). An international study on the diagnostic accuracy of the Japan Narrow-Band Imaging Expert Team classification for colorectal polyps observed with blue laser imaging. Digestion.

[23] Wang L, Lin XC, Li HL (2019). Clinical significance and influencing factors of linked color imaging technique in real-time diagnosis of active Helicobacter pylori infection. Chin Med J (Engl).

[24] Yamasaki Y, Takenaka R, Hori K (2015). Tolerability of magnifying narrow band imaging endoscopy for esophageal cancer screening. World J Gastroenterol.

